# Utilization of provider-initiated HIV testing and counselling in Ethiopia: a systematic review and meta-analysis

**DOI:** 10.1186/s41182-022-00420-9

**Published:** 2022-04-18

**Authors:** Daniel Atlaw, Biniyam Sahiledengle, Sisay Degno, Ayele Mamo, Zewudie Gudisa, Demisu Zenbaba, Zerihun Shiferaw, Habtamu Gezahegn

**Affiliations:** 1 Department of Biomedical Sciences, Madda Walabu University Goba Referral Hospital, Bale-Goba, Ethiopia; 2 Department of Public Health, Madda Walabu University Goba Referral Hospital, Bale-Goba, Ethiopia; 3 Department of Public Health, Madda Walabu University Shashemene Campus, Shashemene, Ethiopia; 4 Department of Pharmacy, Madda Walabu University Goba Referral Hospital, Bale-Goba, Ethiopia; 5 Department of Anesthesia, Madda Walabu University Goba Referral Hospital, Bale-Goba, Ethiopia

**Keywords:** Provider, Initiated, HIV, Testing, Counselling, Meta-analysis

## Abstract

**Background:**

Provider-initiated HIV testing, and counseling (PITC) is a service in which health professionals provide HIV testing to all patients in health facilities. Provider-initiated HIV testing, and counseling is an important opportunity for early screening of individuals, and it is fundamental for both HIV treatment and prevention. Although there are studies conducted in different parts of Ethiopia, their findings are variable. Therefore, this systematic review and meta-analysis aimed to summarize the pooled utilization of PITC in Ethiopia.

**Method:**

All studies conducted on utilization of provider-initiated HIV testing and counseling at outpatient departments (OPD), inpatient departments (IPD), antenatal clinic care (ANC), and tuberculosis (TB) clinics in Ethiopia are eligible for these meta-analyses. A systematic search of the literature was conducted by the authors to identify all relevant primary studies. The databases used to search for studies were PubMed, Science Direct, POPLINE, HENARI, Google Scholar, and Scopus. The extracted data were imported into STATA version 14 software for statistical analysis. The risk of bias was assessed using the Joana Briggs Institute (JBI) criteria for prevalence studies. The heterogeneity among all included studies was assessed by *I*^2^ statistics and the Cochran’s *Q* test. Pooled utilization along with its corresponding 95% CI was presented using a forest plot.

**Result:**

About 1738 studies were retrieved from initial electronic searches using international databases and Google, and a total of 10,676 individual clients were included in the meta-analysis. The pooled utilization of PITC in Ethiopia using the random effects model was estimated to be 78.9% (95% CI 73.87–83.85) with a significant level of heterogeneity (*I*^2^ = 98.5%; *P* < 0.001). Subgroup analysis conducted on PITC showed the highest percentage among studies conducted in Addis Ababa (93.5%), while lower utilization was identified from a study conducted in the Tigray Region (35%).

**Limitation of the study:**

The drawbacks of this review and meta-analysis were being reported with significant heterogeneity, and the protocol was not registered.

**Conclusion:**

About 21% of health facility clients missed opportunities for PITC in Ethiopia.

**Supplementary Information:**

The online version contains supplementary material available at 10.1186/s41182-022-00420-9.

## Background

Provider-initiated HIV testing and counseling (PITC) is a service in which health professionals provide HIV testing to all patients in health facilities [1, 2]. Provider-initiated HIV testing and counseling is an important opportunity for early screening of individuals, and it is fundamental for both HIV treatment and prevention [3, 4]. Routine PITC has the effect of increasing the number of patients tested and, as a result, the number of HIV-positive individuals identified and referred to care and support services [5]. Provider-initiated HIV testing and counseling have increased HIV screening coverage among patients in sub-Saharan Africa [6].

The introduction of PITC by WHO in 2007, although augmented the utilization of HIV screening, has had missed opportunities related to realizing this strategy and variations in acceptance rates [7]. Nevertheless, PITC uses has rapidly increased among pregnant women by its medical care priority, by which it sharply reduced the maternal-to-child HIV transmission rate from 28% in 2009 to 18% in 2013 [8]. In Ethiopia, the maternal-to-child HIV transmission rate was between 11.4% and 9.8% when considering surveys before and after 2012 [9].

All clients visiting health facilities for service at the outpatient department (OPD), inpatient department (IPD), antenatal clinic care (ANC), and tuberculosis clinic (TB) are target populations for PITC in Ethiopia. PITC has developed significantly in recent years in other therapeutic settings, including inpatient, outpatient, and tuberculosis clinics, despite its roots in ANC [10]. Screening through PITC is important for all individuals attending health facilities as HIV will suppress the immune system, predisposing them to different infections. For instance, TB–HIV co-infection was 25% in Ethiopia [11].

Although data are available for different sites of Ethiopia, no nationwide study assessed the pooled proportion of PITC, and the findings are variable [10, 12–28]. For instance, 35% in Tigray [13] and 98% in Bishoftu town [24]. Therefore, this systematic review and meta-analysis aimed to summarize the pooled acceptance rate of PITC in Ethiopia. The findings from this meta-analysis will help policymakers and concerned bodies act on associated factors and thereby increase the utilization of PITC in Ethiopia. This will benefit clients to get early HIV care-related services and reduce the transmission rate of HIV.

## Methods

### Study design and reporting

We conducted review and meta-analysis to estimate the pooled utilization of PITC in Ethiopia among clients of OPD, IPD, ANC, and TB clinics. In this review and meta-analysis, we followed the Preferred Reporting Items for Systematic Reviews and Meta-Analysis (PRISMA) reporting guidelines (Additional file 1).

### Eligibility criteria

*Population* All studies that reported on the PITC utilization rate among clients of OPD, IPD, ANC, and TB clinics.

*Publication status* All published and unpublished articles are considered.

*Year of publication* All publications reported until January 25, 2022, are considered.

*Exclusion criteria* Studies that have reported (voluntary counselling and testing) VCT were excluded from the study.

### The outcome of this systematic review and meta-analysis

Utilization of PITC among clients of OPD, IPD, ANC, and TB clinics.

### Search strategy

The databases used to search for studies were PubMed, Science Direct, POPLINE, HENARI, Scopus, and Google Scholar. Nonpublished articles like thesis and dissertation were searched on Ethiopian university repositories. The final search was updated on January 25, 2022. The following key search terms and medical subject headings [MeSH] were used: “utilization” OR “acceptance” AND “Provider” AND “initiated” AND “HIV testing” AND “counseling” AND “Ethiopia [MeSH]” (Additional file 2). Moreover, the reference lists of the retrieved studies were also scanned to access additional articles and screened against our eligibility criteria.

### Study selection

In this meta-analysis, the duplicate articles were removed, and screening of retrieved articles was conducted independently by two authors (DA and BS) based on the eligibility criteria. The two authors independently screened all retrieved articles, and any disagreement between them was resolved through consensus. Afterwards, full-text articles were retrieved and appraised to determine their eligibility. Finally, the screened articles were compiled together by the two investigators.

### Risk of bias assessment

The qualities of the included studies were assessed, and the risk of bias was judged using the Joanna Briggs Institute (JBI) quality assessment tool for the prevalence studies. Two authors (DA and BS) independently assessed the quality of included studies. Any variation in the final risk of bias assessment among the two authors was declared by discussing on prespecified criteria. The evaluation tool comprises nine parameters: (1) appropriate sampling frame; (2) correct sampling technique; (3) acceptable sample size; (4) study subject and location explanation; (5) appropriate data investigation; (6) use of valid methods for the identified conditions; (7) valid measurement for all participants; (8) using appropriate statistical analysis, and (9) adequate response rate [29]. In this study, since the criteria from number 5 to number 8 were similar for most component studies, we used criteria numbers 1 to 4 to ascertain the risk of bias status. Failure to satisfy each parameter was scored as 1 if not 0. The risks for biases were classified as either low (total score, 0–1), moderate (total score, 2), or high (total score, 3–4) (Additional file 3).

### Data extraction

Two authors (DA and BS) independently extracted data from all included articles and any disagreement was resolved by discussion. The data extraction tool consists of the name of the author(s), year of publication, region, study design, study setting, study population, sample size, utilization with 95% confidence interval, and risk of bias.

### Statistical methods and analysis

The extracted data were imported to STATA version 14 software for statistical analysis. The heterogeneity among all included studies was assessed by I^2^ statistics and the Cochran’s *Q* test. *I*^2^ test statistics results of 25%, 50%, 75%, and 100% are declared as of little concern, concerning, significant, and substantial heterogeneity, respectively. In this meta-analysis, the tests indicate the presence of significant heterogeneity among included studies (*I*^2^ = 98.5%, *P*-value < 0.001). Hence, the heterogeneity is significant and 25 studies are included; we used the random effects model for the final analysis [30, 31]. Subgroup analysis was conducted using the region of the primary study, year of publication, year of study, the population, and risk of bias. A meta-regression analysis was used to evaluate the association between the utilization, and publication year, year of study, and sample size in the selected studies. In this meta-analysis, the authors used funnel plots and Begg’s test to check the presence of publication bias at a significance level of less than 0.05. We have conducted a sensitivity analysis to check for a single study's effect on the overall utilization of PITC.

## Results

### Description of included studies

**Fig. 1:**
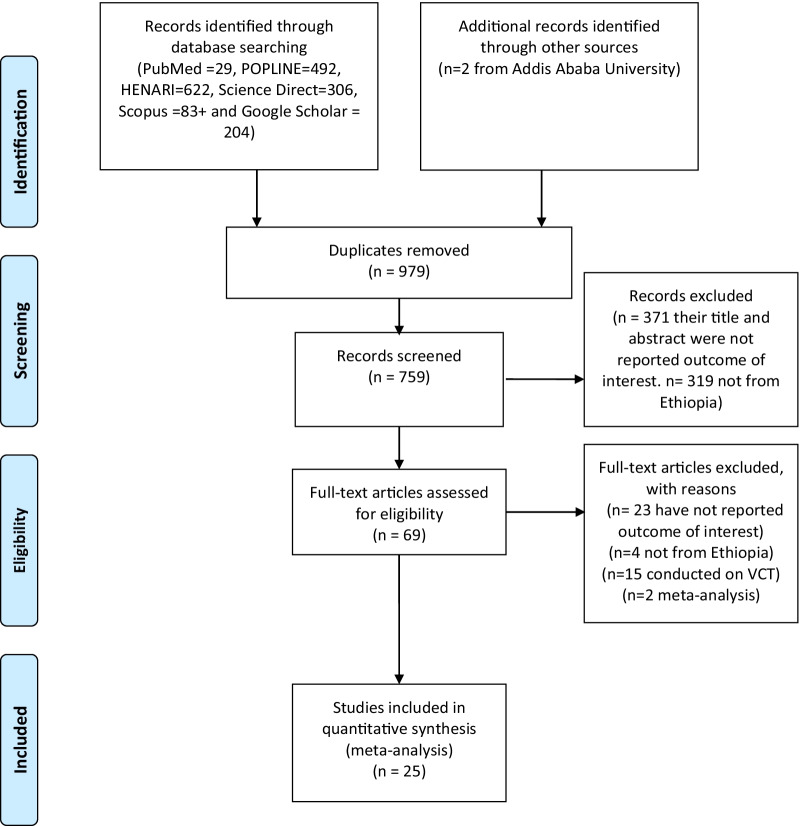
Flow diagram of systemic review and meta-analysis conducted on utilization of PITC Ethiopia, 2021

About 1738 studies were retrieved from initial electronic searches using international databases and Google. The database included PubMed (*n* = 29), HENARI (*n* = 622), POPLINE (*n* = 492), Science Direct (*n* = 306), Google Scholar (*n* = 204), Scopus (*n* = 83), and Addis Ababa University repository (*n* = 2) studies. Of these, 979 duplicates were removed, and the remaining 759 articles were screened, and 690 articles were excluded after reading their titles and abstracts. Sixty-nine full-text articles remained and were further assessed for their eligibility. Finally, based on the pre-defined inclusion and exclusion criteria, 25 articles were included in the meta-analysis, and data were extracted for the final analysis (Fig. 1).

### Characteristics of the included studies

A total number of 10,676 individual clients were included in the meta-analysis. All included studies are cross-sectional and health facility-based. The latest article was conducted in 2019, and the earliest study was concluded in 2009. Depending on sample size, 17 studies have a sample size greater than or equal to 384, and eight studies less than 384 participants. Ten studies were conducted in the Oromia region, five in the Amhara region, three studies in the Southern Nation and Nationality Region (SNNR), two studies in the Harari region, two studies in the Benishangul region, and one study in each of the Tigray and Afar regions. About four studies were conducted on TB clients, six studies were conducted among OPD clients, and fifteen studies were conducted among ANC clients. Concerning the risk of bias among studies, most studies were judged as having a high or moderate risk of bias (Table 1).

**Table 1:** Characteristics of included studies for pooled utilization of PITC in Ethiopia

Author name	Year of publication	Region	Year of study	Setting	Study design	Population	Response rate	Mean age of respondents	Sampling technique	Sample size	PITC utilized	Utilization in %	Confidence interval	Risk of bias
Hasen	2009	Oromia	2009	Facility based	Cross-sectional	ANC client	100	24.24	Systematic random sampling	422	370	87.7	84.56–90.84	Low
Tesfaye et al.	2010	Oromia	2010	Facility based	Cross-sectional	OPD client	92.3	Not reported	Cluster sampling	539	448	81	76.86–85.14	Moderate
Deribew et al.	2011	Oromia	2011	Facility based	Cross-sectional	TB client	not reported	Not reported	Not clear	506	298	58.9	53.86–62.14	Moderate
Hailu et al.	2011	Amhara	2011	Hospital based	Cross-sectional	OPD client	83.6	25	Not clear	411	346	83.6	79.95–87.25	Moderate
Mohammed et al.	2011	SNNP	2011	Facility based	Cross-sectional	ANC client	100	Not reported	Not clear	326	218	66.9	61.73–72.04	High
Addisu	2012	Benishangul	2012	Facility based	Cross-sectional	OPD client	91.5	30	Systematic random sampling	384	315	80.9	85.43–76.37	High
Malaju et al.	2012	Amhara	2010	Facility based	cross-sectional	ANC client	97.6	25.37	Simple random sampling	400	330	82.5	78.78–86.22	Moderate
Araya	2013	Tigray	2009	Hospital based	Cross-sectional	OPD client	99.3	33.2	Convenience sampling	415	145	35	30.41–39.59	Low
Yadeta et al.	2013	Oromia	2011	Hospital based	Cross-sectional	TB client	99.9	38.4	Survey	681	669	98	96.94–99.06	Low
Adeba et al.	2014	Oromia	2010	Facility based	Cross-sectional	TB client	100	Not reported	Quota sampling	237	213	89.9	85.88–93.92	Low
Deresa et al.	2014	Addis Ababa	2010	Facility based	Cross-sectional	ANC client	99.5	25.4	Simple random sampling	734	686	93.5	91.83–95.17	Low
Kebede et al.	2014	Oromia	2010	Facility based	Cross-sectional	TB client	100	35	Simple random sampling	399	353	88.5	86.42–90.58	Low
Abdurahman et al.	2015	Harari	2011	Facility based	Cross-sectional	OPD client	98.6	Not reported	Systematic random sampling	513	362	70.6	68.52–72.68	High
Abtew et al.	2015	Benishangul	2014	Facility based	cross-sectional	ANC client	94.6	31.9	Systematic random sampling	386	312	80.8	76.88–84.72	Moderate
Fikadu D.et al	2016	Oromia	2013	Facility based	Cross-sectional	OPD client	98.6	28.2	Systematic random sampling	371	291	78.4	74.13–82.67	High
Gebermedihin et al.	2016	Oromia	2016	Facility based	Cross-sectional	ANC client	100	Not reported	Not clear	441	309	70.1	65.83–74.37	High
Merga H.et al.	2016	Oromia	2015	Facility based	Cross-sectional	ANC client	99.2	25.2	Not clear	374	325	86.9	83.35–90.45	Moderate
Alemu et al.	2017	Amhara	2012	Facility based	Cross-sectional	ANC client	98.6	28	Systematic random sampling	416	277	67	62.83–71.17	High
Akal et al.	2018	Afar	2015	Facility based	Cross-sectional	ANC client	100	Not reported	Not clear	347	246	70.9	66.12–75.68	High
Bekele et al.	2018	Harari	2018	Facility based	Cross-sectional	ANC client	100	Not reported	Simple random sampling	278	229	82.4	77.93–86.87	Moderate
Gizaw et al.	2018	SNNP	2017	Facility based	Cross-sectional	ANC client	98.6	24	Systematic random sampling	504	424	84.1	80.91–87.29	Moderate
Geberesellase et al.	2019	Amhara	2019	Hospital based	Cross-sectional	ANC client	89.8	26	Systematic random sampling	364	330	90.7	86.23–95.17	Low
Gebeyehu et al.	2019	SNNP	2019	Facility based	Cross-sectional	ANC client	96	27.9	Systematic random sampling	340	234	68.8	63.81–73.74	High
Zegeye et al.	2020	Amhara	2019	Facility based	Cross-sectional	ANC client	99.1	30.7	Stratified sampling	355	308	86.8	83.27–90.33	High
Anbese et al.	2021	Oromia	2018	Facility based	Cross-sectional	ANC client	100	Not reported	Systematic random sampling	354	302	85.3	81.36–89.24	Moderate

### Utilization of PITC Ethiopia

**Fig. 2:**
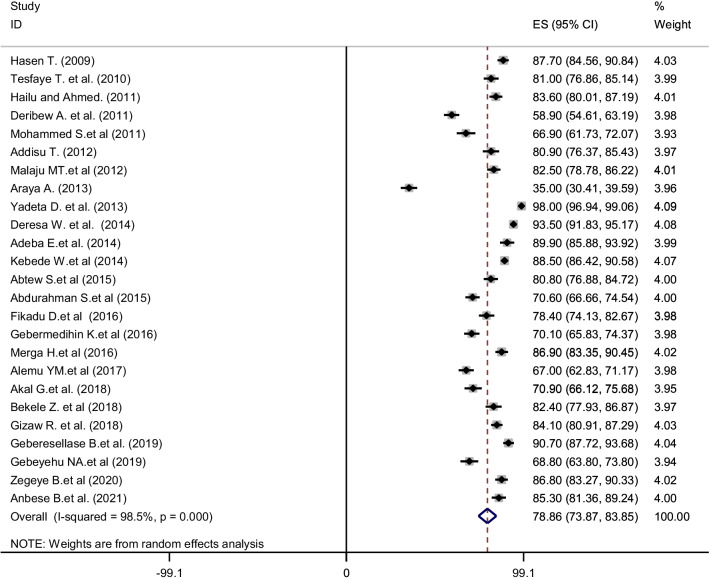
Forest plot showing pooled utilization of provider-initiated HIV testing and counselling in Ethiopia

The pooled utilization of PITC in Ethiopia using the random effect model was estimated to be 78.9% (95% CI 73.87–83.85) with a significant level of heterogeneity (*I*^2^ = 98.5%; *P* < 0.001) (Fig. 2). Subgroup analysis was conducted by year of publication, year of study, region, the population of the study, and risk bias of studies. The pooled utilization of PITC was 72% (95% CI 60.40–83.82) among studies published between 2009 and 2012. Among studies published between 2013 and 2016, it was 84% (95% CI 78.17–90.28). The heterogeneity was shown to vary from 95.0% to 98.1% in this subgroup analysis.

Sub-group by year of the study conducted revealed 78% (95% CI; 70.69–85.99) among studies conducted between 2009 and 2012, while 83.2% (95% CI 78.08–88.37) among studies conducted between 2017 and 2019.

Subgroup analysis conducted on PITC found that the highest utilization was among studies conducted in Addis Ababa (93.5%), while lower utilization was identified from a study conducted in the Tigray Region (35%). In this subgroup, the heterogeneity varied from 0% in the Benishangul region to 98% in the Oromia region.

The pooled utilization of PITC was identified to be 84.5% (95% CI 73.41–93.57) among high-risk of bias studies and 71.9 (95% CI 66.71–77.34) among low-risk of bias studies. The heterogeneity was shown to decrease from 99% to 14.7% after subgroup analysis by the risk of bias (Table 2).

**Table 2:** Sub-group analysis by region, year of publication, year study, study population and risk of bias on pooled utilization of PITC in Ethiopia

Sub-group analysis	Number of studies	Utilization of PITC	95% confidence interval	Heterogeneity (I^2^%)	*P*-value
Sub-group analysis by year of publication					
1. 2009–2012	8	72.1	60.40–83.82	98.5	*P* < 0.001
2. 2013–2016	9	84.2	78.17–90.28	98.1	*P* < 0.001
3. 2017–2021	8	79.6	73.47–84.20	95.0	*P* < 0.001
Sub-group analysis by year of study					
1. 2009–2012	13	78.34	70.69–85.99	99.0	*P* < 0.001
2. 2013–2016	6	75.75	69.35–82.15	93.1	*P* < 0.001
3. 2017–2019	6	83.23	78.08–88.37	92.1	*P* < 0.001
Sub-group analysis by regions of Ethiopia					
1. Amhara region	5	82.2	74.81–89.58	95.4	*P* < 0.001
2. Oromia	10	82.5	76.16–90.34	98.4	*P* < 0.001
3. Harari	2	76.5	64.89–88.01	93.4	*P* < 0.001
4. Tigray	1	35.0	30.41–39.59	–	–
5. Benishangul	2	80.8	77.88–83.81	0.0	*P* = 0.987
6. Afar	1	79.9	66.12–75.68	–	–
7. Addis Ababa	1	93.5	91.83–95.17	–	–
8. Southern Nation Nationality Region of Ethiopia	3	73.4	61.49–85.31	95.4	*P* < 0.001
Sub-group population					
1. TB clinic clients	4	84.0	71.42–96.48	99.1	*P* < 0.001
2. ANC clients	15	80.4	75.84–85.04	96.2	*P* < 0.001
3. OPD clients	6	71.6	57.84–85.37	98.5	*P* < 0.001
Sub-group analysis by risk bias of studies					
1. High	9	84.5	73.41–93.57	99.0	*P* < 0.001
2. Moderate	9	83.5	82.04–84.92	14.7	*P* = 0.315
3. Low	7	71.9	66.71–77.34	93.5	*P* < 0.001

### The publication biases

**Fig. 3:**
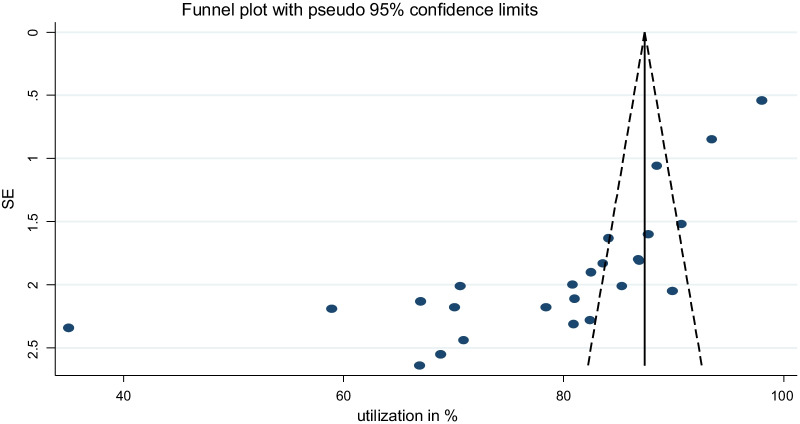
Funnel plot showing publication bias states of studies included for pooled analysis of provider-initiated HIV testing and counselling in Ethiopia

**Fig. 4:**
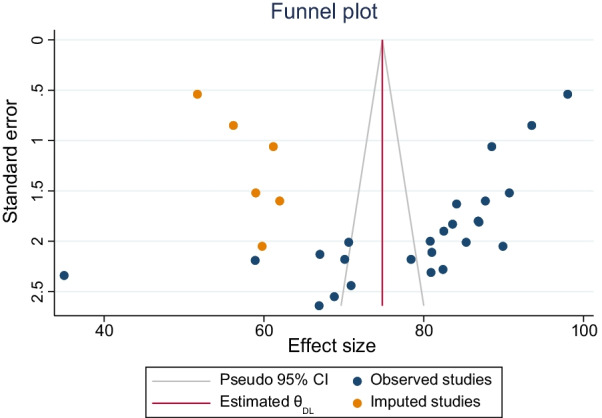
Showing studies added by the trim-and-fill analysis of provider-initiated HIV testing and counselling in Ethiopia

The presence of publication bias was evaluated using funnel plots and Begg’s tests at a significance level of less than 0.05. The findings revealed that publication bias was significant for the studies on the utilization of PITC in Ethiopia (*P* < 0.001) (Fig. 3). The fill-and-trim analysis added six studies, and the pooled utilization was decreased to 74.2% (Fig. 4).

### Sensitivity analysis

**Fig. 5:**
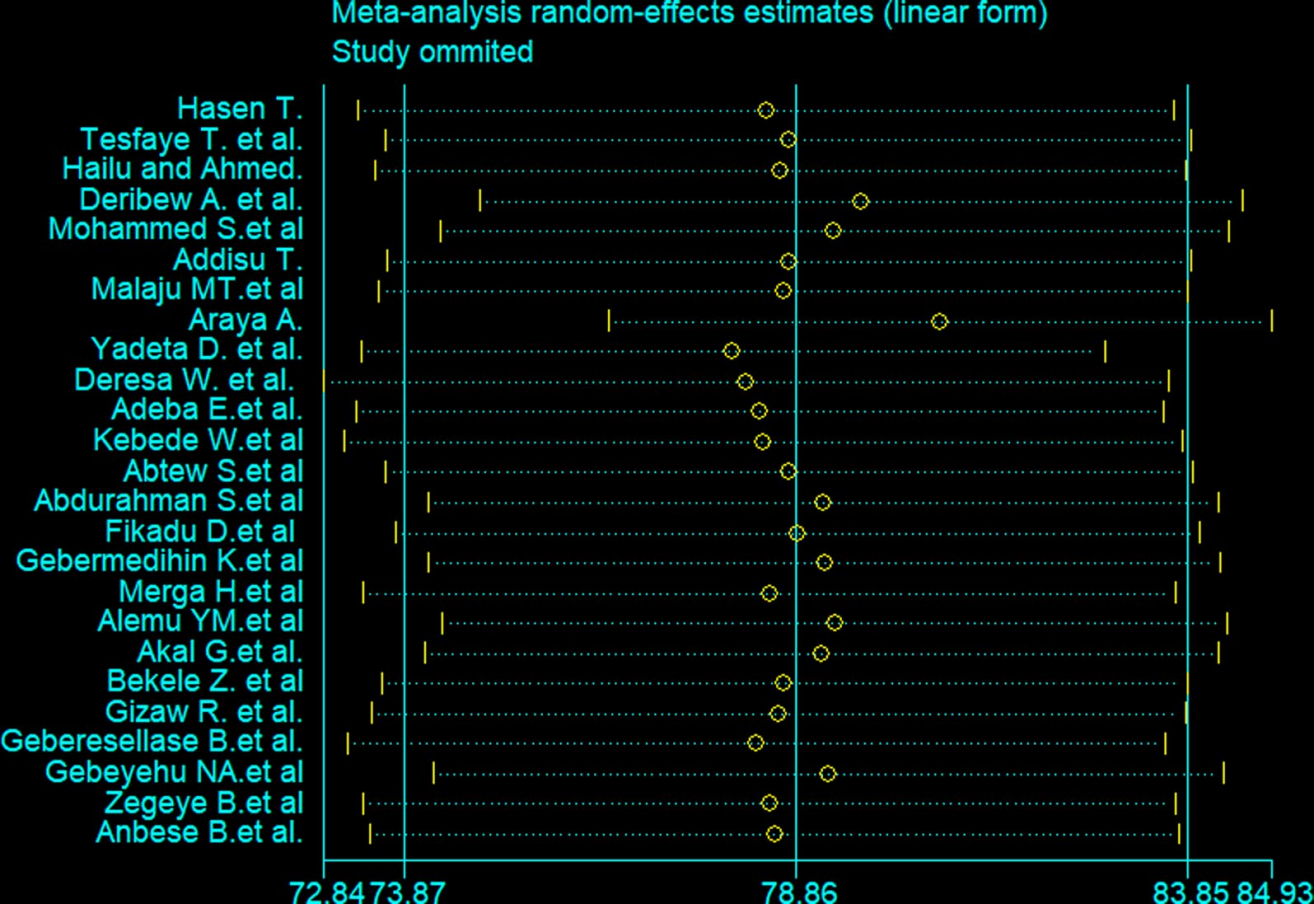
Sensitivity analysis of studies included for pooled utilization provider initiated HIV testing and counselling in Ethiopia

The presence of a single study effect on pooled utilization of PITC is tested using meta influence analysis and revealed that there was no single study that significantly affected pooled utilization of PITC in Ethiopia (Fig. 5).

### Meta-regression

In a meta-regression analysis, the publication year, study year, and sample size were not significant sources of heterogeneity for the utilization of PITC among clients of health facilities in Ethiopia. In this study, no significant relationship was identified between utilization of PITC and the year of study (*P*-value = 0.48), the publication year (*P*-value = 0.64), and sample size (*P*-value = 0.44).

## Discussion

This meta-analysis is the first pooled utilization of PITC conducted among clients of health facilities in Ethiopia. The utilization level of PITC was revealed to be 78.9% in Ethiopia. Still, pooled utilization identified in this review was lower than the WHO target of 2020 [32]. Further, the finding of this meta-analysis was lower than the data from Nigeria, which was 93% [33]. The difference might be due to sociodemographic differences between the two countries. Further, defective supply chains on HIV commodities like HIV test kits may play a role in the differences in the utilization level of PITC. For instance, in the southern region of Ethiopia, the stockout rate of HIV/AIDS commodities was about 53.3% [34]. In Addis Ababa, about 75% of health facilities were revealed to have at least one and above stockout of HIV commodities. This shows that stockout may contribute to lower utilization of PITC in Ethiopia [35].

Subgroup analyses by the year of component studies have shown a slightly higher utilization of PITC between 2017 and 2019 than those conducted earlier. The variation may be explained by the implementation of different HIV prevention program that focuses on screening service to achieve the 2020 WHO target. For instance, priority population targeted testing was in 2017 by International Center for AIDS Care and Treatment Programs (ICAP) [36].

Subgroup analysis from this meta-analysis shows the highest utilization was identified among studies conducted in Addis Ababa, while the lowest utilization was identified among studies conducted in the Tigray region of Ethiopia. Variation in the knowledge of HIV/AIDS may be one of the reasons for the difference in utilization levels among different regions of Ethiopia [37]. For instance, comprehensive HIV knowledge was identified to be 42.5% in Addis Ababa, while it was lower in the other regions and administrative towns of Ethiopia [38]. Further, the capacity score for HIV/AIDS service declines as we go to the remote zones of regions in Ethiopia [39]. The difference can also be explained by the difference in universal health service coverage among different regions and administrative towns of Ethiopia [40].

The subgroup analysis identified higher PITC utilization among TB clinic clients, while lower utilization was among OPD clients. Indeed, PITC is mandatory for TB clinic clients in Ethiopia given their higher risk of HIV infection, 25% in Ethiopia [11].

This meta-analysis revealed that high-risk-of-bias studies had a higher PITC utilization rate which can be attributable to the fact that these studies likely overestimated the pooled effect size and was evidenced by its wide confidence interval [41].

### Limitation and strength of the study

This meta-analysis is the first nationwide study to pool the PITC utilization rate in Ethiopia. Still, it has several limitations. First, the *I*^2^ value, as over 75% here, suggested substantial heterogeneity among the included studies. This could be explained by the differences in geographical locations and clinical settings, in that the PITC utilization rates were pooled. Finally, the protocol of this study was not registered on PROSPERO, which is a pitfall of the systematic review.

## Conclusion

The utilization of PITC is lower when compared with the 2020 target set by WHO. From our finding, about 21% of health facility clients may have missed opportunities for PITC in Ethiopia. Therefore, we recommend further national studies to identify factors that contribute to the lower utilization level of PITC in Ethiopia. In addition, further initiation of the health facilities to achieve the recommended target of 2025 by WHO, thereby increasing screening and early treatment to reduce complications caused by HIV infection and related comorbidities.

## Supplementary Information

**Additional file 1.** PRISMA checklist for systematic review and meta-analysis on utilization of PITC in Ethiopia.

**Additional file 2.** Supplementary file showing search strategies for different database on utilization of PITC in Ethiopia.

**Additional file 3.** Risk of bias assessment of studies included for systematic review and meta-analysis on utilization of PITC in Ethiopia.

## Data Availability

The part of the data analyzed during this study is included in this manuscript. Other data will be available from the corresponding author upon a reasonable request.
