# Supplementary material for: Utilization of provider-initiated HIV testing and counselling in Ethiopia: a systematic review and meta-analysis
Source: Trop Med Health. 2022 Apr 18;50:29. doi: 10.1186/s41182-022-00420-9 (PMC9016945; doi:10.1186/s41182-022-00420-9)

**Supplementary file showing search terms and data base searched for systematic review and meta-analysis on utilization of PITC in Ethiopia.**

**PubMed** **Search**: **(((((providers) AND (initiated)) AND (HIV)) AND (testing)) AND (counseling)) AND (Ethiopia)**

("provide"[All Fields] OR "provided"[All Fields] OR "provider"[All Fields] OR "provider s"[All Fields] OR "providers"[All Fields] OR "provides"[All Fields] OR "providing"[All Fields]) AND ("initial"[All Fields] OR "initially"[All Fields] OR "initials"[All Fields] OR "initiate"[All Fields] OR "initiated"[All Fields] OR "initiates"[All Fields] OR "initiating"[All Fields] OR "initiation"[All Fields] OR "initiations"[All Fields] OR "initiator"[All Fields] OR "initiators"[All Fields]) AND ("hiv"[MeSH Terms] OR "hiv"[All Fields]) AND ("test s"[All Fields] OR "tested"[All Fields] OR "testing"[All Fields] OR "testings"[All Fields] OR "tests"[All Fields]) AND ("counsel"[All Fields] OR "counseled"[All Fields] OR "counselings"[All Fields] OR "counselled"[All Fields] OR "counselling"[All Fields] OR "counseling"[MeSH Terms] OR "counseling"[All Fields] OR "counsellings"[All Fields] OR "counsels"[All Fields]) AND ("ethiopia"[MeSH Terms] OR "ethiopia"[All Fields] OR "ethiopia s"[All Fields])


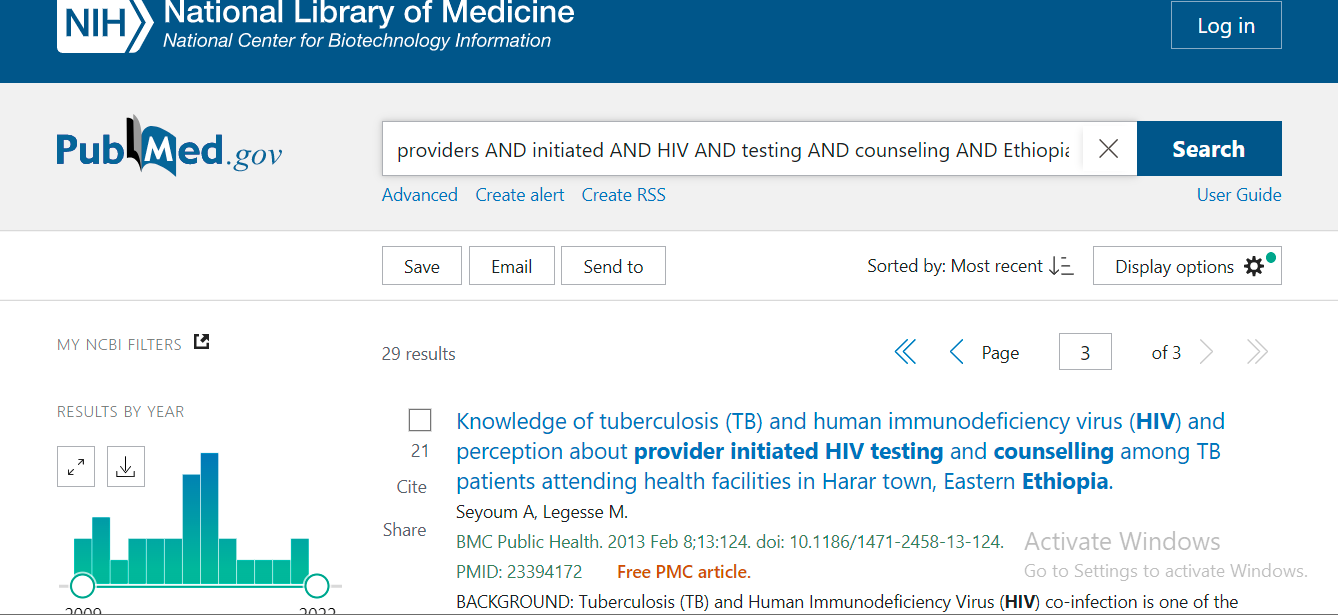


**HENARI Search**: **(((((providers) AND (initiated)) AND (HIV)) AND (testing)) AND (counseling)) AND (Ethiopia)**


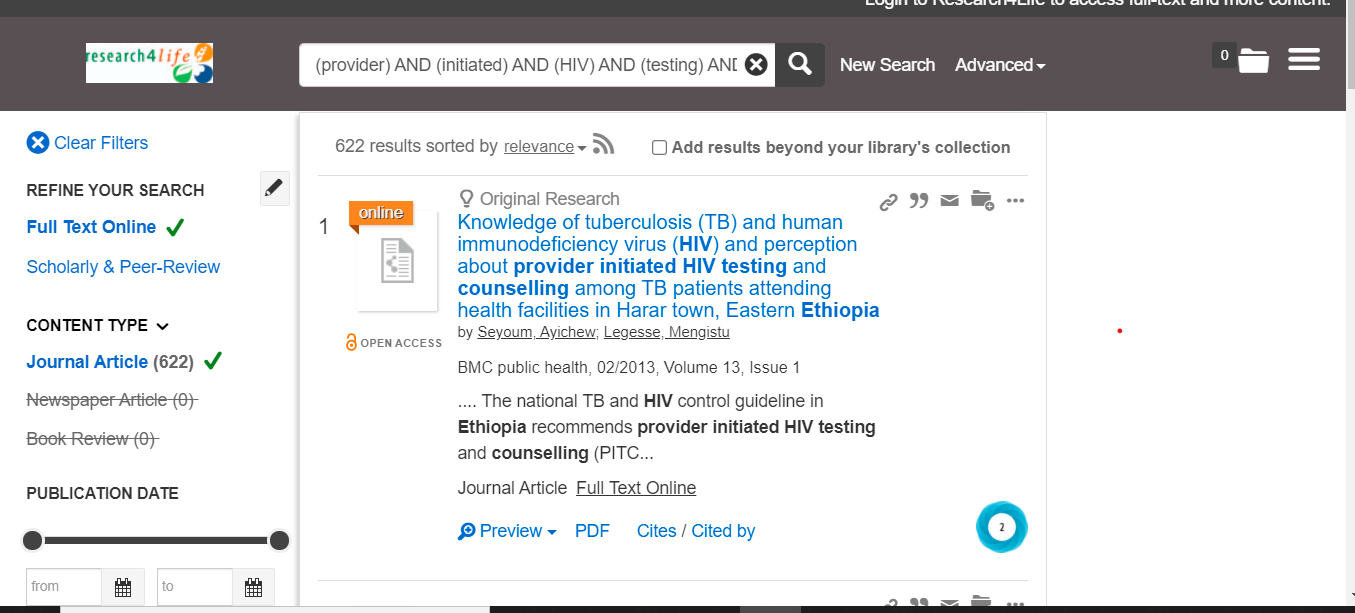


**POPLINE Search**: **(((((providers) AND (initiated)) AND (HIV)) AND (testing)) AND (counseling)) AND (Ethiopia)**


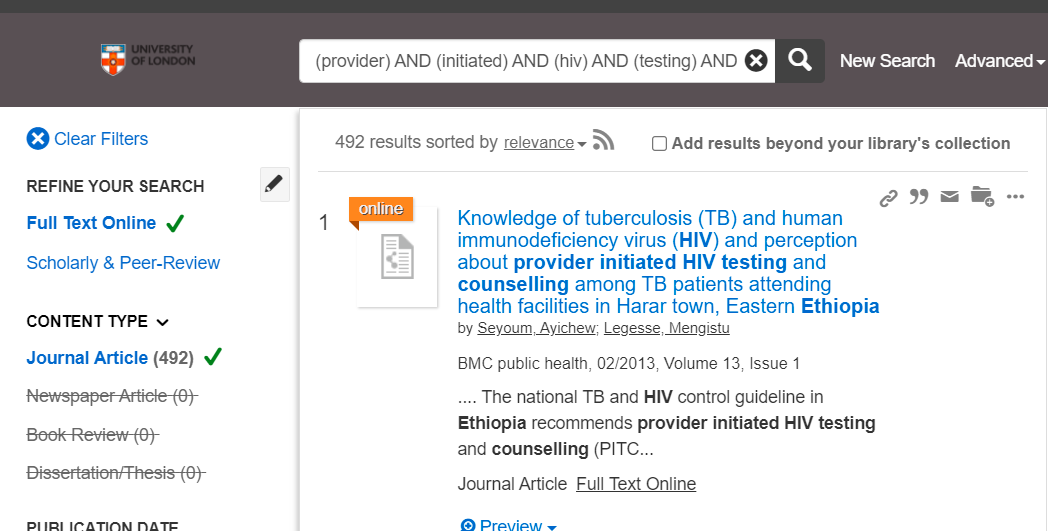


**SCIENCE DIRECT Search**: **(((((providers) AND (initiated)) AND (HIV)) AND (testing)) AND (counseling)) AND (Ethiopia)**


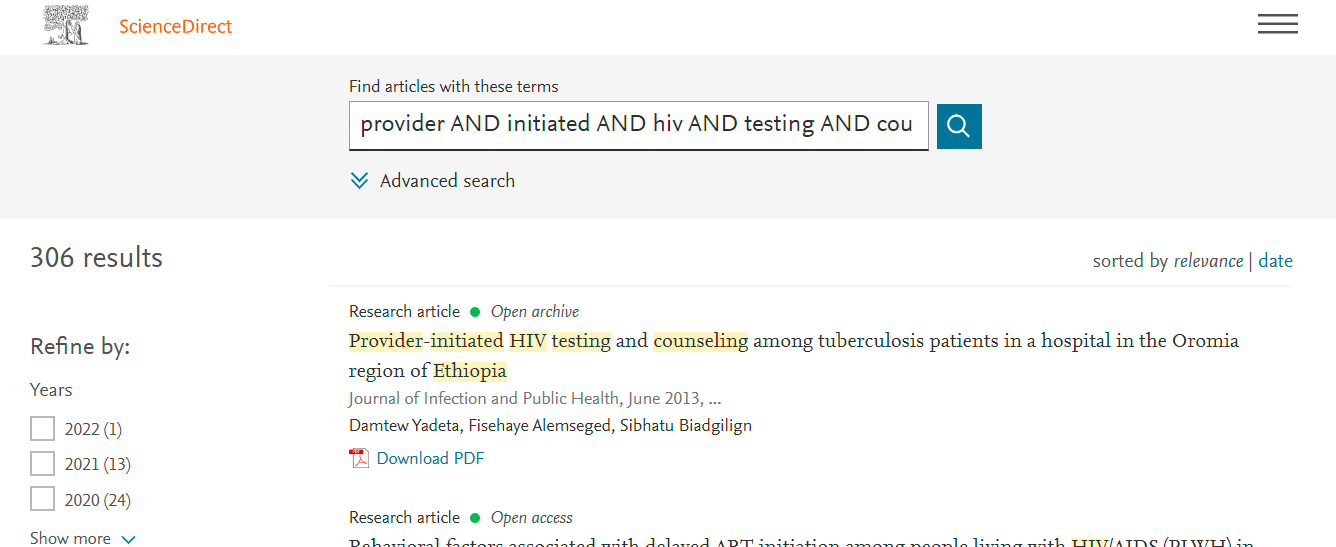


**Search on Google Scholar**

**Termes used -Utilization, OR acceptance AND "provider initiated HIV testing and counselling" AND Ethiopia - "Voluntary counselling and testing"**


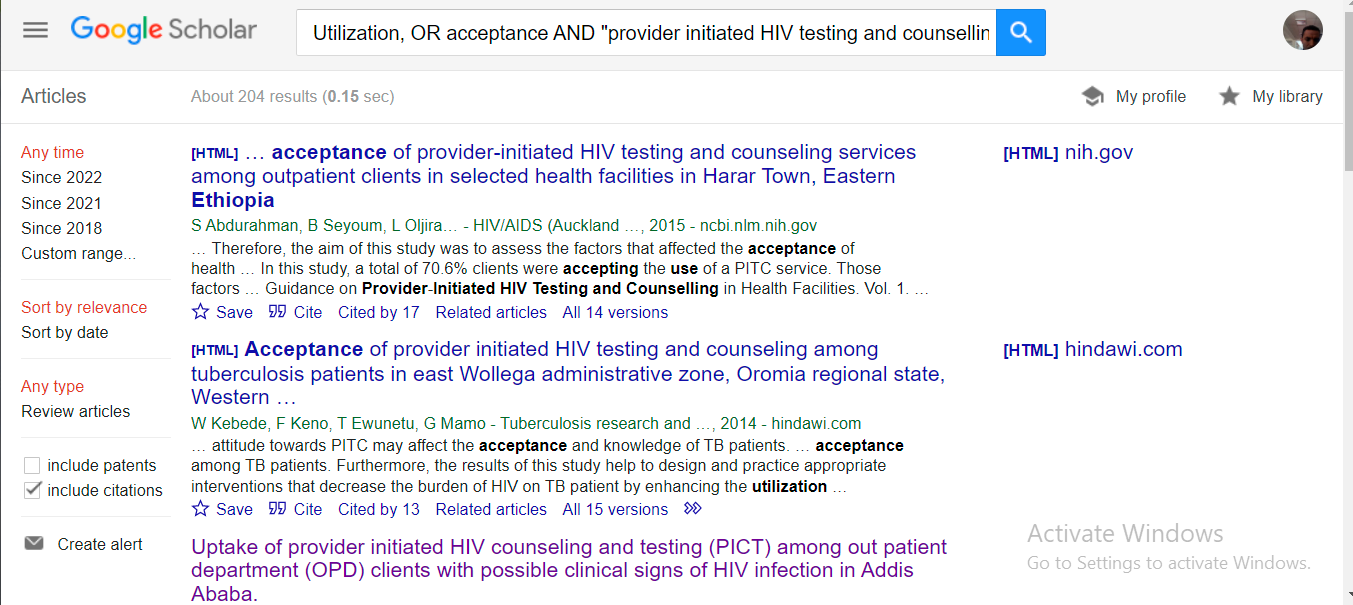


SCOPUS

provider AND initiated AND HIV AND testing AND counselling AND Ethiopia AND ( LIMIT-TO ( AFFILCOUNTRY , "Ethiopia" ) )


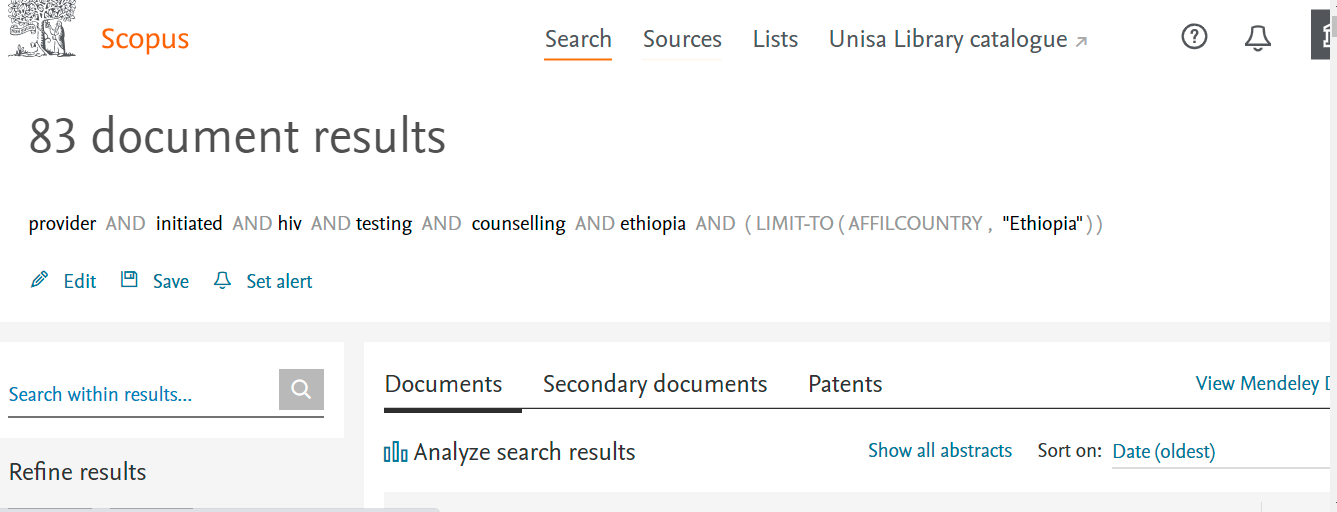


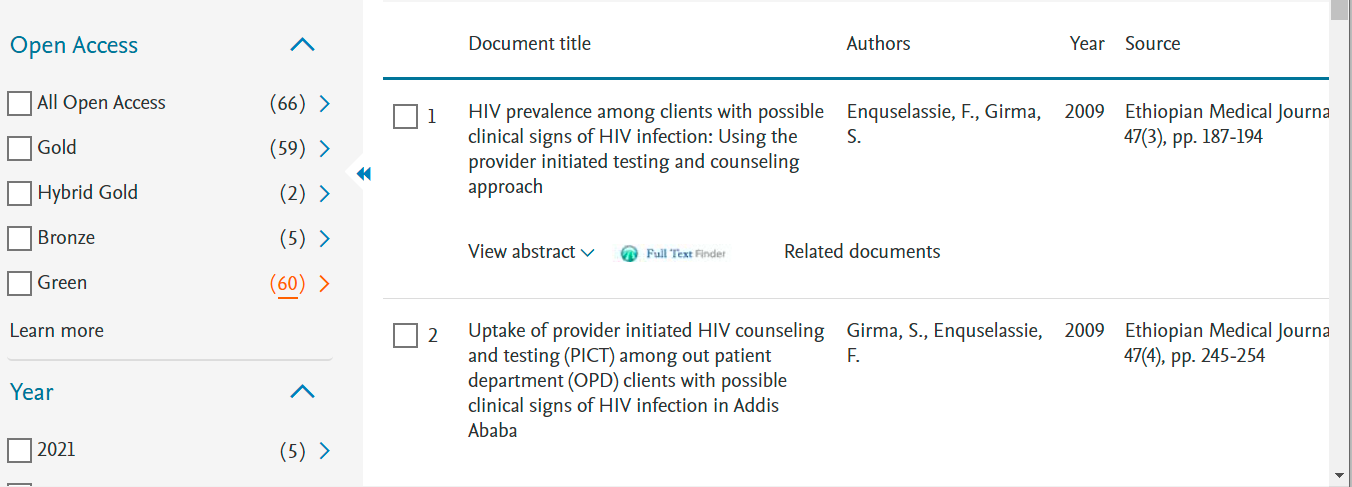

Supplement: Supplementary file 2 — Additional file 2. Supplementary file showing search strategies for different database on utilization of PITC in Ethiopia. [file 41182_2022_420_MOESM2_ESM.docx]
